# Perception of lower back pain associated with use of body armor in police officers of the countryside specialized battalion of Ceará, Brazil

**DOI:** 10.47626/1679-4435-2023-809

**Published:** 2023-04-18

**Authors:** Evanice Avelino de Souza, João Paulo da Silva Albuquerque, Felipe Rocha Alves, Carlos Antônio Dias Ferreira

**Affiliations:** 1 Educação Física, Faculdade Terra Nordeste, Fortaleza, CE, Brazil; 2 Departamento de Ciências Médicas, Universidade Federal do Ceará, Fortaleza, CE, Brazil

**Keywords:** police, occupational health, work, lower back pain, polícia, saúde do trabalhador, trabalho, dor lombar

## Abstract

**Introduction:**

The strict demands of the military environment, associated with the increase in violence, as well as the frequent use of body armor, can further aggravate health problems.

**Objectives:**

To investigate the perception of police officers of the Countryside Specialized Police Battalion in relation to comfort, fatigue, and lower back pain, resulting from the use of body armor.

**Methods:**

This was a cross-sectional study conducted with 260 male military police officers (34.62 ± 5.83 years old) belonging to the ostensive rural police battalion in the state of Ceará, Brazil. The questionnaire related to comfort, fatigue, and lower back pain was used to identify the perception of pain from the use of body armor, with staggered responses, and the results were analyzed using the SPSS 21.0 software.

**Results:**

Regarding the use of body armor, 41.5% of participants perceived it to be little comfortable in general; furthermore, 45 and 47.5% of military police officers considered it little comfortable in relation to weight and use during operational activities, respectively. With regard to body measurements, 48.5% reported being little comfortable, and 70% perceived that the body armor is adjustable to the body. At the end of the work shift, 37.3% complained of lower back pain, and 45.8% felt moderate fatigue. Moreover, 70.1% felt pain in the lower back after the work shift.

**Conclusions:**

Military police officers reported lower back pain at the end and after the work shift due to use of body armor, as well as little comfort of the protective equipment and moderate fatigue at the end of the work shift.

## Abstract (pt)

**Introdução:**

As rigorosas exigências do ambiente militar associadas ao aumento da violência, bem como ao uso frequente do colete balístico, podem agravar ainda mais os problemas de saúde.

**Objetivos:**

Investigar a associação do uso de colete balístico em relação ao conforto, à fadiga e à queixa de lombalgia em policiais do batalhão especializado do Ceará.

**Métodos:**

Tratou-se de um estudo transversal realizado com 260 policiais militares do sexo masculino (34,62 ± 5,83 anos) pertencentes ao batalhão de policiamento ostensivo rural do estado do Ceará. O questionário relacionado a conforto, fadiga e dores na região lombar foi utilizado para identificar a percepção de dor pelo uso do colete balístico, com respostas escalonadas, e seus resultados foram analisados no *software* SPSS 21.0.

**Resultados:**

Quanto ao uso do colete, 41,5% perceberam ser pouco confortável no geral; e 45 e 47,5% dos policiais militares consideraram ser pouco confortável em relação ao peso e em relação ao uso durante as atividades operacionais, respectivamente. No que se refere às medidas corporais, 48,5% reportaram ser confortável, e 70% percebem o colete como ajustável ao corpo. No momento da finalização do turno de trabalho, 37,3% apresentam dores na região lombar e 45,8% sentem fadiga moderada. Após o encerramento do turno de trabalho, 70,1% sentem dores na região lombar.

**Conclusões:**

Os policiais militares perceberam dores na região lombar ao final e depois do turno de trabalho pelo uso do colete balistico, além de relatarem sobre o pouco conforto do equipamento de proteção e sobre a fadiga moderada ao fim do turno de trabalho.

## INTRODUCTION

According to the Ministry of Health,^1^ occupations with a high level of occupational stress, responsibilities, and risks are more prone to develop physical and mental health-related problems, including the following professionals: physicians, nurses, teachers, journalists, and especially military police officers.

The military police is one of the professional categories in which exposure to risks for physical integrity is evident. The strict demands of the military environment, associated with the increase in violence and lack of preparation or of professional conditions and personal support, required for good professional performance, make the military police one of the most stressful jobs.^2-4^

In addition to these factors, the frequent use of body armor, weaponry, ammunition, gun belt, uniform, and handcuffs^5^ can further aggravate health problems. One of most important health-related aspect of military police officers is physical pain resulting from conditions to which they are exposed during work.^6,7^

However, health-related aspects of these professionals and their needs have been having little visibility and social understanding, with a small scientific production on this issue in Brazil and other countries from Latin America.^7^ The first Brazilian research started in 2020 and, according to the authors, aimed to investigate health and sickness of public safety agents in the state of Ceará, Brazil. In addition to performing blood pressure and glucose measurement, nutritional follow-up, ergonomic assessment, and provision of postural guidelines appropriate to the workplace, this research promotes integrative practices such as massages, auriculotherapy, and medicinal tea, due to the scarcity of studies offering a comprehensive view of police officers.^8^

However, existing investigations specifically assessed stress and resilience,^9^ stress and quality of life,^10^ diabetes mellitus,^11^ cardiovascular changes,^12^ and metabolic syndrome and quality of life.^13^ However, few national studies aimed to analyze physical pain from use of work equipment among military police officers. In view of this gap, we aimed to investigate the perception of military police officers of Countryside Specialized Police Battalion (Batalhão Especializado de Policiamento do Interior, BEPI) in relation to comfort, fatigue, and lower back pain from use of body armor.

## METHODS

This was a quantitative, cross-sectional study conducted from September to October 2020, in the 4^th^ BEPI. This BEPI includes military police officers working at Rural Tactical Command (Comando Tático Rural, COTAR) and Border Police Organization (Companhia de Policiamento de Divisas, CPD), responsible for high-risk ostensive policing in rural areas to inhibit the action of criminal groups involved in the robbery of banks and/or armored cars, support countryside police officers and to more serious situations, and support to police officers from regions that share a border with the state of Ceará to inhibit the advance of the New Cangaço (a movement that includes groups of outlaws acting in Northeastern Brazil).

Military police officers were approached during working hours and invited to voluntarily participate, being informed about the study and its predicted benefits, with guarantees of confidentiality, privacy, and anonymity. The study was submitted to the Research Ethics Committee of Faculdade Terra Nordeste (FATENE) and approved by opinion no. 21924019.5.0000.8136. The research complied with the human research standards set forth in resolutions no. 466/2012 and 510/2016.

**Figure 1:**
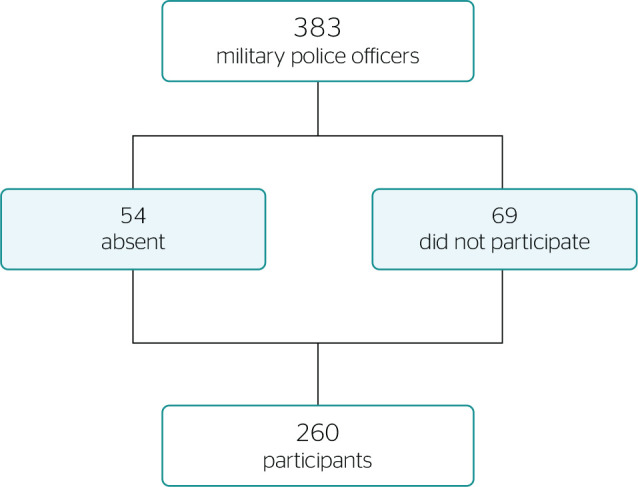
Description of population and sample of the present study.

According to the directing committee of the battalion, there were 383 police officers. However, 54 were absent for several reasons. Therefore, the 329 professionals who were actively working at the time of the study were invited to participate in the research. Nonetheless, 69 did not participate in the study because there were changes in the work schedule on the day of data collection. All participants signed the informed consent form (ICF) (Figure 1).

In order to investigate participants’ perception about comfort regarding use of body armor, they were asked about the comfort of the armor regarding weight, use during operational activities, and body measures. Options of answer were subdivided into extremely comfortable, uncomfortable, little comfortable, comfortable, and very uncomfortable. With regard to armor adjustment to the body, the options of answer were non-adjustable, almost any adjustment, little adjustable, adjustable, very adjustable, and extremely adjustable.

Perception of pain at the dorsolumbar region was also investigated, and respondents could report unbearable pain, intense pain, moderate pain, little pain, and no pain. Furthermore, it was assessed whether military police officers felt fatigue due to use of the armor, with the possible options of answer: unbearable fatigue, intense fatigue, moderate fatigue, mild fatigue, and no fatigue at the end of the work shift.

The instrument used was based on the study by Santos et al.,^7^ conducted with military police officers, due to the lack of pre-existing research protocol translated, transculturally adapted, and validated to Brazilian Portuguese.

Military police officers were also asked whether they felt body pain after using the body armor, i.e., after their work shift. If they answered “yes,” they were asked to indicate the painful region with an “x” on a human body drawing. It was possible to indicate more than one region.

Sociodemographic variables included age, duration of employment, schooling (complete secondary school, incomplete higher education, complete higher education, and graduate education), place of work (COTAR and CPD), and practice of physical activity outside work (no or yes).

Initially, data normality was assessed by Kolmogorov-Smirnov test, descriptive analysis was conducted using the SPSS 21.0 software, and results were expressed as absolute and percentage frequencies, mean and standard deviation.

## RESULTS

Overall, 260 military police officers of specialized units in the state of Ceará, Brazil, participated in the study, with mean age of 34.62 ± 5.83 years. Mean duration of employment was 3.05±2.71 years. With regard to schooling, 35.4% had complete secondary education; furthermore, 50% worked at COTAR; and 60.8% reported to perform physical activity outside work, as shown in Table 1.

**Table 1:** Sociodemographic characteristics of police officers of Countryside Specialized Police Battalion (Batalhão de Policiamento Especializado do Interior, BEPI) in Ceará, Brazil (2020)

Variables	Mean (± SD)	
Continuous		
Age (years)	34.62 ± 5.83	
Duration of employment (years)	3.05 ± 2.71	
Categorical	n	%
Schooling		
Complete secondary education	92	35.4
Incomplete higher education	76	29.2
Complete higher education	76	29.2
Graduate education	15	5.8
Place of work		
COTAR	130	50.0
CPD	130	50.0
Engagement in PA		
Yes	158	60.8
No	102	39.2

COTAR = Rural Tactical Command (Comando Tático Rural); CPD = Border Police Company (Companhia de Policiamento de Divisas); PA = physical activity; SD = standard deviation.

Regarding use of the body armor, 41.5% perceived it to be little comfortable in general; and 45 and 47.5% of military police officers considered it little comfortable regarding weight and regarding wearing the armor during operational activities, respectively. As for the item related to comfort with the armor regarding body measures, 48.5% reported it was comfortable, and 70% thought the armor was adjustable to the body. At the end of the work shift, 37.3% complained of lower back pain, and 45.8% felt moderate fatigue, as presented in Table 2.

**Table 2:** Perception of police officers of Countryside Specialized Police Battalion (Batalhão de Policiamento Especializado do Interior, BEPI) in Ceará, Brazil (2020) regarding comfort with use of the body armor

Variables	n	%
Comfort/discomfort with the armor		
Extremely uncomfortable	3	1.2
Uncomfortable	47	18.1
Little comfortable	108	41.5
Comfortable	96	36.9
Very uncomfortable	6	2.3
Comfort/discomfort regarding weight of the armor		
Extremely uncomfortable	6	2.3
Uncomfortable	66	25.4
Little comfortable	117	45.0
Comfortable	60	23.1
Very uncomfortable	11	4.2
Comfort/discomfort regarding wearing the armor during operational activities		
Extremely uncomfortable	3	1.2
Uncomfortable	61	23.6
Little comfortable	123	47.5
Comfortable	63	24.3
Very uncomfortable	9	3.5
Comfort/discomfort with the armor regarding body measures		
Extremely uncomfortable	4	1.5
Uncomfortable	29	11.2
Little comfortable	93	35.8
Comfortable	126	48.5
Very uncomfortable	8	3.1
Armor adjustment to the body		
Non-adjustable	5	1.9
Almost any adjustment	3	1.2
Little adjustable	52	20.0
Adjustable	184	70.8
Very adjustable	15	5.8
Extremely adjustable	1	0.4
Pain at dorsolumbar region at the end of the work shift		
Unbearable pain	3	1.2
Intense pain	12	4.6
Moderate pain	97	37.3
Little pain	96	36.9
Without pain	52	20.0
Fatigue at the end of the work shift wearing the armor		
Unbearable fatigue	1	0.4
Intense fatigue	19	7.3
Moderate fatigue	119	45.8
Mild fatigue	90	34.6
No fatigue	31	11.9

**Figure 2:**
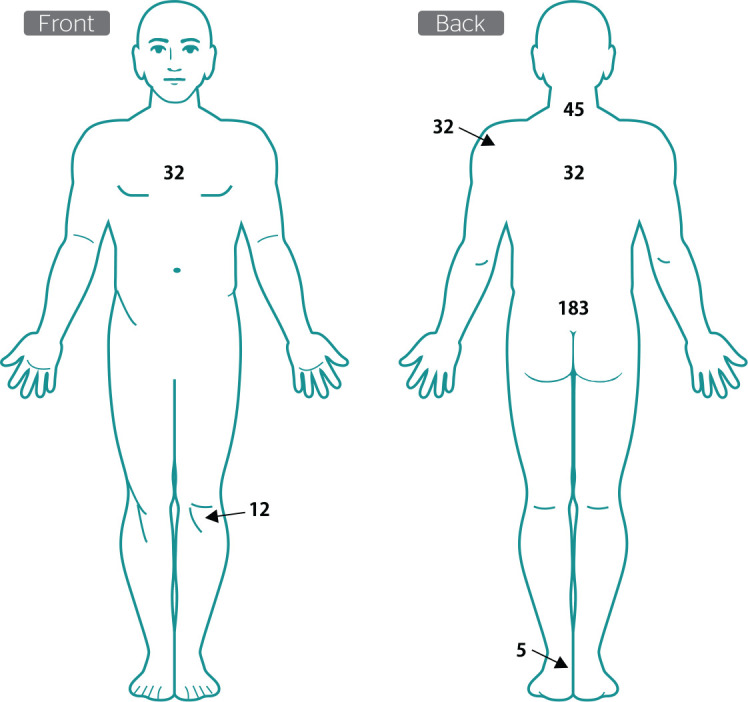
Frequency (n) of perceived pain after the work shift among police officers of Countryside Specialized Police Battalion (Batalhão de Policiamento Especializado do Interior), in Ceará, Brazil (2020).

All military police officers complained of body pain after their work shift; most of them (183; 70.1%) complained of lower back pain. Figure 2 shows the other body regions reported as painful.

## DISCUSSION

So far, this is the first study conducted with military police officers of BEPI that aimed to investigate the perception of these professionals regarding comfort, fatigue, and lower back pain due to use of body armor. The main outcome of the present research was the prevalence of lower back pain at the end and after the work shift. Our findings may be corroborated by a recent systematic review aiming to investigate the frequency of musculoskeletal symptoms in different parts of the body in military police officers. The results of this study showed that the frequency of musculoskeletal symptoms within 12 months ranged from 42-52% for the lower back, the body region reported to be the most affected by pain. The authors reported that, although police officer is an occupation that exists worldwide, with a significant number of workers, few specific studies on the musculoskeletal health of this population are found in the literature.^14^

It is believed that one of the causes for the prevalence of low back pain is officers’ bad posture while they remain inside the police vehicle, which, according to their work routes, may vary from 4 to 8 hours, with short intervals during the two work shifts.^15^ A study conducted with Canadian police officers of both sexes found high levels of discomfort in the lumbar, sacrum, and pelvis regions after an 8 hour work shift. This discomfort was related to the use of gun belt, armament, and body armor, and to the police vehicle seat.^16^ This condition intensifies compressive load on the intervertebral disc, generating fatigue of erector spinae muscles, which should be activate to maintain a straight sitting posture.^17^

Another hypothesis to justify this result are movements (jump over a wall, approaching people, driving and running at a high speed) that are extremely physically demanding and require a good level of capability during patrol, with the emergence of police occurrences - situations in which police officers, in addition to perform the aforementioned movements, wear personal protective equipment (PPE), such as body armor and gun belt, and adopt inappropriate postures during working hours.^18^

In this sense, it would be interesting to implement a physical exercise program guided by a professional in the area, aiming to promote muscle strengthening of abdominal, lumbar, pelvic, and hip regions, since both absence and excess of physical effort may cause damages to individuals’ mechanics.^6,19^ Importantly, an intervention study conducted in Curitiba, state of Paraná, Brazil, with 42 male military police officers and that, among other objectives, aimed to investigate the effect of adopting Fowler’s position during 20 minutes at the end of the work shift on recovering stature and observed a significant stature recover among military police officers. The author recommends adopting recovery postures, such as Fowler’s position (lying supine position dorsal with the legs supported on a bench), to prevent and/or reduce spine-related diseases.^20^ Furthermore, educational activities to promote postural reeducation at work and massage services could be available for military police officers at battalions before and/or after the work shift.

Concerning the degree of comfort/discomfort with use of body armor, most participants reported that it is little comfortable and also little comfortable regarding weight and use during service. These results are consistent with those found in the literature.^6,21-24^ A study that aimed to perform an ergonomic analysis of body armors used by 89 police officers of 2^nd^ Organization of the 4^th^ Paraná’s Military Police Battalion, using technical data from the equipment and application of perception questionnaires found a need for improvement in some characteristics of this equipment. Among them, there changes in equipment weight, size, and flexibility, in addition to improvement in body temperature balance while wearing the equipment in order to improve professional performance.^25^

According to the National Institute of Justice (NIJ), the armor should follow specifications based on types, models, and sizes. However, constant and inappropriate use of armors, together with extensive work hours, may contribute to a greater perception of discomfort with use body armors, especially because they impair movements and increase locomotion time, possibly compromising the performance of military functions.^7^

The statistical laboratory of the Federal Bureau of Investigation (FBI) showed that, of the 1,708 polices officers feloniously killed in the line of duty from 1987 to 2015, 1,574 were killed by firearms. However, more than 3,100 police officers have been saved from death or serious injury by wearing body armor.^26^ Therefore, public policy programs to educate military police officers on the importance of wearing the aforementioned equipment are extremely important, in addition to the development of investigation to monitor their perception about this equipment, as well as possible adjustments to make it more comfortable.

The military police officers in the present study also reported feeling moderate fatigue after the work shift from use of the armor. It is worth highlighting that this feeling of fatigue reported by participants may also be added to characteristics of their occupation, since these professionals spend part of their time in the state’s countryside and away from their families, in addition to environmental conditions of state’s countryside (high temperatures) and the fact that officers need to pay for their own food during on-duty days.

A study conducted with 39 police officers of both sexes of 1^st^ Radio Patrol Company of 4^th^ Battalion in Guarabira, state of Paraíba, Brazil, reported that fatigue had a significant influence on quality of life and may also be considered a risk factor for reduced working capacity.^27^ Considering that the work developed by military police officers in the present study became an international benchmark in policing of rural areas,^28^ it is interesting to maintain accommodations with the required conditions for officers’ physical and mental rest, with promotion of playful and sports activities, as well as high-speed internet for a better communication with family members.

In the present study, military police officers complained of pain in the neck, shoulders, back, and knees after use of body armor. A recent study conducted with 55 military police officers of both sexes of the garrison of 15^th^ Battalion of Patos de Minas, state of Minas Gerais, Brazil, found that complaints of musculoskeletal pain from use of body armor were reported in different body regions after the work shift.^29^ A systematic review study that aimed to describe the frequency of musculoskeletal symptoms at different body regions among police officers also found a high frequency of musculoskeletal symptoms among military police officers, especially at the lumbar, dorsal, knee, neck, and shoulder regions.^30^ Hence, there is a need for further investigations aimed at detecting pain-related factors in order to propose strategies to improve military police officers’ quality of life, because pain may be aggravated by use of armor, but are associated with other factors.

Some issues may be considered as study limitations: (1) application of questionnaires after lockdown caused by the novel coronavirus pandemic, which prevented greater participation; (2) lack of a validated instrument to assess comfort perception with use of body armor; and (3) sample consisting only of men. However, the results found can be useful for future interventions, due to the scarcity of studies on this theme, allowing for police unit commanders to search for strategies that respond to police officers’ needs, promoting a better quality of life at work.

## CONCLUSIONS

This research found reports of perceived pain at the end of the work shift due to use of body armor and after the work shift, in addition to obtaining reports on little comfort of this protective equipment and on moderate fatigue at the end of the work shift.

In view of what was shown about a still little explored theme, our results may provide noticeable contributions to health research, especially in the occupational health field. Therefore, it is worth emphasizing the importance of the development of actions by healthcare professionals, especially of the physical education and physiotherapy, in order to foster health promotion at work, as well as activities of prevention and recovery from damages related to the use of body armor.

The present study is expected to have contributed to advances in information about the health of military police officers from the 4^th^ Battalion in the state of Ceará, Brazil, as well as to future interventions aiming to improve quality of life in the work environment of these professionals.
